# Electrophysiological and behavioral evidence for attentional up-regulation, but not down-regulation, when encoding pictures into long-term memory

**DOI:** 10.3758/s13421-018-0871-z

**Published:** 2018-10-19

**Authors:** Christopher S. Sundby, Geoffrey F. Woodman, Keisuke Fukuda

**Affiliations:** 1 Department of Psychology, Vanderbilt Vision Research Center, Center for Integrative and Cognitive Neuroscience, Vanderbilt University, Nashville, TN USA; 2 Department of Psychology, University of Toronto Mississauga, 3359 Mississauga Rd North, Mississauga, ON L5L 1C6 Canada

**Keywords:** Voluntary control, Visual long-term memory encoding, EEG

## Abstract

Visual long-term memory allows us to store a virtually infinite amount of visual information (Brady, Konkle, Alvarez, & Oliva in *Proceedings of the National Academy of Sciences of the United States of America, 105*(38), 14325–14329, 2008; Standing in *Quarterly Journal of Experimental Psychology, 25*(2), 207–222, 1973). However, our ability to encode new visual information fluctuates from moment to moment. In Experiment 1, we tested the hypothesis that we have voluntary control over these periodic fluctuations in our ability to encode representations into visual long-term memory using a precueing paradigm combined with behavioral and electrophysiological indices of memory encoding. We found that visual memory encoding can be up-regulated, but it was much more difficult, if not impossible, to down-regulate encoding on a trial-by-trial basis. In Experiment 2, we tested the hypothesis that voluntary up-regulation of visual memory encoding for an item incurs a cost to memory encoding of other items by manipulating the cueing probability. Here, we found that, although the cueing benefit was constant for both low (20%) and high (50%) cueing probabilities, the benefit in the high cueing probability condition came with the overall impairment of memory encoding. Taken together, our findings demonstrate that top-down control of visual long-term memory encoding may be primarily to prioritize certain memories, but this prioritization has a cost and should not be overused to avoid its negative consequences.

Long-term memory allows us to store vast, if not unlimited, amounts of information in our mind for later retrieval (Brady, Konkle, Alvarez, & Oliva, 2008; Standing, 1973). However, our ability to fully utilize this large storage capacity is constrained by moment-to-moment fluctuations in our ability to encode new information (Fernández et al., 1999; Fukuda & Woodman, 2015; Noh, Herzmann, Curran, & de Sa, 2014; Paller & Wagner, 2002; Wagner et al., 1998). This cognitive constraint significantly impacts the quality of our daily lives considering the amount of visual information that we have to remember for later use (e.g., where I last saw my phone or what groceries to buy on the way home after work). Specifically, can we voluntarily up-regulate our memory encoding when we encounter information that we need to remember? On the other hand, we occasionally encounter information that we do not wish to remember (e.g., a spoiler of a movie you are planning to watch this weekend). In such situation, can we down-regulate our memory encoding so that we will not remember the information later?

Studies of voluntary attentional control have demonstrated that we are capable of enhancing the information processing of task-relevant information while suppressing the processing of task-irrelevant information (Gaspelin, Leonard, & Luck, 2015, 2017; Hickey, Di Lollo, & McDonald, 2009; Noonan et al., 2016; Noonan, Crittenden, Jensen, & Stokes, 2017; Posner, 1980; Sawaki & Luck, 2011). Given a number of studies revealing a close relationship between attentional control and memory encoding (e.g., Chun & Turk-Browne, 2007; deBettencourt, Norman, & Turk-Browne, 2017; Moray, 1959; Turk-Browne, Golomb, & Chun, 2013; Uncapher, Hutchinson, & Wagner, 2011), it is reasonable to hypothesize that we can enhance (or *up-regulate*) and degrade (or *down-regulate*) our ability to encode visual long-term memory.

Previous studies show preliminary support for this hypothesis. For example, individuals can remember information better if its successful encoding promises a larger amount of reward (Adcock, Thangavel, Whitfield-Gabrieli, Knutson, & Gabrieli, 2006; Gruber & Otten, 2010; Gruber, Watrous, Ekstrom, Ranganath, & Otten, 2013). Although this result seems to indicate that we are capable of up-regulating memory encoding at will, it is not clear whether the anticipation of external reward is necessary to exert such voluntary control.

In addition, other lines of work demonstrated that people remember items that were cued to be forgotten more poorly than those cued to be remembered. More precisely, in typical item-method directed-forgetting paradigms, participants are presented with a cue to remember or forget the immediately preceding item, and such studies have consistently found that items that were followed by the “remember” cue are better remembered than items followed by “forget” cues (e.g., Anderson & Hanslmayr, 2014; Bjork, Bjork, & Anderson, 1998; MacLeod, 1998, 1999). Although these findings have often been interpreted as evidence for individuals’ ability to intentionally forget unwanted information, an alternative explanation is that individuals are selectively up-regulating the encoding of the cued-to-be-remembered items compared with the cued-to-be-forgotten items. To test this, it is important to test the memory performance for cued-to-be-remembered and cued-to-be-forgotten items against the “baseline” items that did not receive either of the cues while keeping other factors equal. However, the majority of directed forgetting studies, except for a few recent studies (Gao et al., 2016; Taylor, Quinlan, & Vullings, 2018; Zwissler, Schindler, Fischer, Plewnia, & Kissler, 2015), had not performed this comparison due to the lack of within-subject baseline conditions. Therefore, we directly addressed this issue in Experiment 1.

In addition, in the directed-forgetting literature, it is considered best practice to provide instructional cues *after* the perceptual encoding of the stimulus in order to equate the attentional allocation at the time of perceptual encoding for cued-to-remember and cued-to-forget items. However, in the present study, we wanted to study just that: With front-end attentional mechanisms at our disposal, can we boost the encoding of some memories while diminishing the encoding of others. As a result, we provided the voluntary cues prior to the perceptual encoding of the stimulus. This way, we maximized the possibility to observe our ability to voluntarily up-regulate and down-regulate memory encoding via front-end attentional mechanisms.

## Experiment 1

In Experiment 1, we examined whether individuals can voluntarily up-regulate and down-regulate memory encoding in response to a cue provided prior to the onset of the stimulus. Here, we utilized three types of cues in addition to baseline trials in which no cue was provided. One cue instructed participants to try extra hard to encode the cued stimulus (i.e., *up-regulation*). Another cue instructed participants to try not to remember the cued stimulus (i.e., *down-regulation*). A third cue instructed participants to try to encode the cued stimulus in the same manner as the baseline trials (i.e., *neutral*). The introduction of this cue allowed us to compare the effect of trial-by-trial voluntary control of memory encoding while controlling for any confound induced by the physical onset of the cue. If it is possible to voluntarily up-regulate memory encoding, then memory performance for cued-to-be-up-regulated items would be better than that for neutral cue items as well as baseline items. On the other hand, if cueing benefits are entirely driven by the bottom-up salience of the cued items, then memory performance for all the cued items will be equally better than that of baseline items because each type of cue was presented with equal frequency. Lastly, if individuals are capable of voluntarily down-regulating memory encoding, the memory performance for cued-to-be-down-regulated items should be worse than that for neutral and baseline items.

To characterize the nature of voluntary control of memory encoding with converging measures, we also examined the previously established electrophysiological correlates of memory encoding success. Specifically, we measured the frontal positivity and the occipital alpha power suppression while participants encoded stimuli into visual long-term memory. The frontal positivity is a sustained positive deflection that onsets approximately 300 ms after the onset of a stimulus to encode. The occipital alpha power suppression is the reduction in the amplitude of alpha frequency activity (i.e., 9–13 Hz) observed over the occipital channels. This amplitude reduction onsets approximately 300 ms after the stimulus onset and is known to persists for several hundred milliseconds. Previous studies have demonstrated that items that are later remembered are encoded with larger frontal positivity (e.g., Friedman & Johnson, 2000; Fukuda & Woodman, 2015) and larger and more sustained occipital alpha power suppression (e.g., Fukuda & Woodman, 2015; Hanslmayr, Staudigl, & Fellner, 2012) than items that are later forgotten. If we can voluntarily modulate memory encoding, we should expect these two correlates of memory encoding success to be modulated in the direction indicated by the cue. More specifically, if we can voluntarily up-regulate memory encoding, then the frontal positivity should be larger and the occipital alpha power should be smaller when participants up-regulate their memory encoding. On the other hand, if we can voluntarily down-regulate memory encoding, then the frontal positivity should be smaller and the occipital alpha power should be larger when participants attempt to down-regulate their memory encoding.

### Method

#### Power calculation

In Experiment 1, we conducted a repeated-measures ANOVA with one within-subjects factor of cue type (baseline, neutral, up-regulation, and down-regulation). Anticipating that we will obtain a moderate effect size of Cohen’s *f* = .25 (Cohen, 1988) of the cue type, the a priori power calculation with an alpha level of .05, the statistical power of .8 and .6 correlation coefficients among the repeated-measures indicated that we would need 23 participants (Faul, Erdfelder, Lang, & Buchner, 2007). This assures that our sample size is sufficient to detect a moderate size effect with .8 statistical power.

#### Participants

All participants gave written informed consent according to procedures approved by the Vanderbilt University Institutional Review Board. All volunteers self-reported that they were neurologically normal, had normal or corrected-to-normal visual acuity, and were not color-blind. Data from three participants were excluded due to excessive oculomotor artifacts (>30% of trials), leaving 24 participants in the sample.

#### Stimuli

The stimuli were adapted from a published set of photographs (Brady et al., 2008) and presented in MATLAB using the Psychophysics Toolbox (Brainard, 1997; Pelli, 1997). Participants were seated approximated 60 cm from the CRT monitor. Cues were presented in the center of the screen and were one of four colors depending on the task and condition (RGB values: *x* = 0.592, *y* = 0.367, 9.60 cd/m^2^; green, *x* = 0.299, *y* = 0.579, 27.6 cd/m^2^; yellow, *x* = 0.396, *y* = 0.509, 35.5 cd/m^2^; black, *x* = 0.393, *y* = 0.423, 0.31 cd/m^2^). Stimuli subtending 11.5 × 11.5 degrees in visual angle were presented on a white background (*x* = 0.293, *y* = 0.323, 38.5 cd/m^2^).

#### Tasks

##### Encoding task

**Fig. 1:**
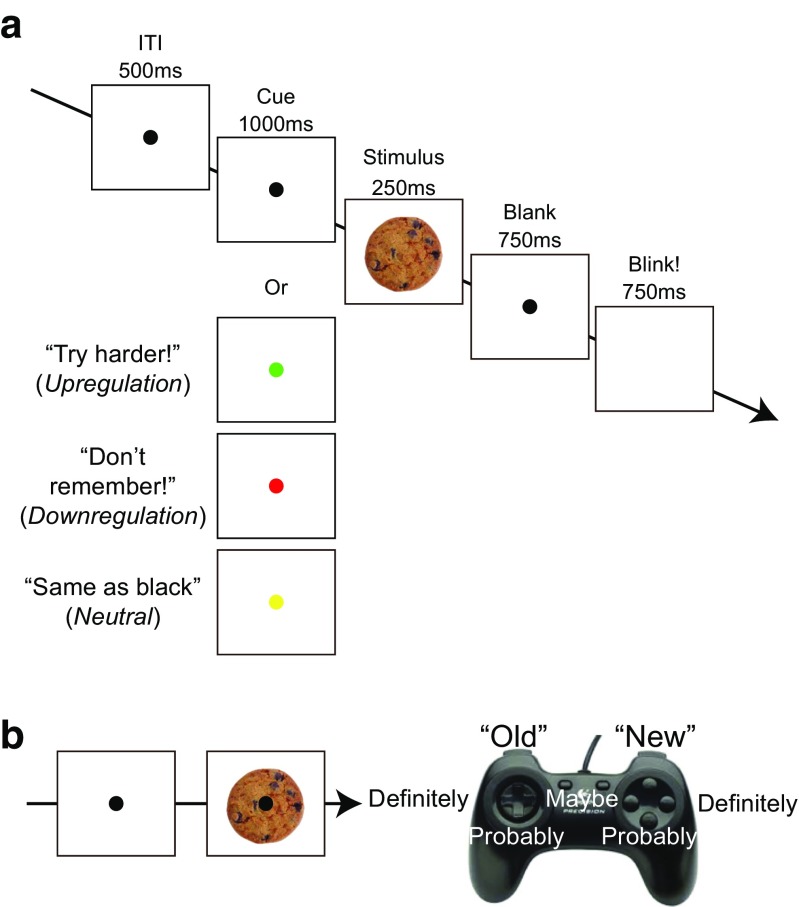
**a** Schematic of encoding task in Experiment 1. **b** Schematic of recognition task in Experiment 1. (Color figure online)

In the first phase of the experiment, participants were sequentially presented with 900 pictures of real-world objects with short breaks every 90 pictures. They were instructed to study each item while holding central fixation so that they could later perform a recognition memory test. Each trial started with a presentation of a black central fixation dot (see Fig. 1). After 500 ms, the central fixation dot turned into one of four cues, as explained below. Following the 1,000-ms-long cue presentation, a picture was presented on the computer screen for 250 ms and then was followed by 750-ms-long blank display. After the blank display, the fixation dot disappeared to allow participants to blink for 750 ms, and the next trial started automatically. The trials of the different conditions were randomly intermixed throughout the encoding task.

During the 600 baseline trials, the black fixation dot remained the same. During the 100 *up-regulation* trials, the fixation dot turned green, and this cue instructed participants to try extra hard to encode the cued stimulus. During the 100 *down-regulation* trials, the central fixation dot turned red, and this cue instructed participants to try not to remember the cued stimulus. During the 100 *neutral* trials, the central fixation dot turned yellow, and this cue instructed participants to try to encode the cued stimulus in the same manner as the *baseline* trials. This condition was critical to control for the stimulus-driven effect that might be observed in the *up-regulation* and *down-regulation* trials due to the change in fixation point color. The colors of cues were fixed across participants to keep the color-to-condition mapping the most intuitive to of all the participants (i.e., green up-regulation and red down-regulation).

##### Recognition task

The recognition memory test started with the onset of a central fixation dot following the break after the encoding task. Participants were instructed to maintain central fixation without blinking until each trial was over. Following the button press, a picture of a real-world object was presented at the center of the screen (new and old pictures were randomly interleaved across trials). Participants were instructed to indicate whether they remembered seeing this picture anytime during the experiment, irrespective of encoding condition, and provided a confidence rating by pressing one of six buttons on the game pad. This ensured that participants had to make recognition judgments based on one criterion across all the items presented (i.e., “Did I see this item during the encoding task or not?), and therefore justified the construction of the encoding-condition-specific receiver operating characteristics (ROC) curves using a common false-alarm rate for the new pictures (e.g., Fukuda & Woodman, 2015; Yonelinas, Dobbins, Szymanski, Dhaliwal, & King, 1996). Three buttons on the right side of the game pad were used to indicate that they did not remember the item, and the three buttons on the left side of the game pad were used to indicate that they did remember the item. Of the three buttons on each side, the outermost indicated 100% confidence (i.e., definitely) in their judgment, the middle button indicated 80% confidence (i.e., probably), and the inner button indicated 60% confidence (i.e., maybe). After the response the trial was over, and participants were provided with a self-determined interval to rest their eyes and blink. Participants were tested on 900 studied pictures and 450 new pictures across 10 blocks. Of note, there were more studied pictures presented than new pictures during the recognition test. This likely induced response bias favoring “remember” responses across the board. Importantly, the number of old items from different encoding conditions were equal and presented randomly, intermixed with new items. Thus, any bias should affect all encoding conditions equally, and therefore does not cause problems for interpreting differences observed among different encoding conditions.

#### EEG data acquisition and analysis

The EEG data were recorded using a right-mastoid reference and were referenced off-line to the average of the left and right mastoids. We used the international 10–20 electrode sites (Fz, Cz, Pz, F3, F4, C3, C4, P3, P4, PO3, PO4, O1, O2, T3, T4, T5, and T6) and a pair of custom sites, OL (halfway between O1 and T5) and OR (halfway between O2 and T6). Eye movements were monitored using electrodes placed 1-cm lateral to the external canthi for horizontal movement and an electrode placed beneath the right eye for blinks and vertical eye movements. The signals were amplified with a gain of 20,000, band-pass filtered from 0.01 to 100 Hz, and digitized at 250 Hz. Trials accompanied by horizontal eye movements (>30 μV mean threshold across observers) or eye blinks (>75 μV mean threshold across observers) were rejected before further analyses. The thresholds for artifact rejection were customized to each participant, with 15.6% of trials rejected on average across participants such that they were not entered into any of the following analyses.

##### ERP analysis

To examine the event-related potentials (ERPs) during memory encoding, we time-locked waveforms to the onset of memory stimuli and examined the ERPs recording from −200 to 1,000 ms following the onset of each memory stimulus. These ERP epochs were baseline corrected to the mean amplitude −200 to zero ms relative to the stimulus onset.

##### EEG analysis

The same artifact-free trial epochs in ERP analysis was subjected to EEG time-frequency analyses. To allow a sufficiently long data segment for oscillatory analyses, we appended a 1,200-ms-long buffer window at each end of the trial epochs. Then, we bandpass filtered each artifact-free trial epoch with 2-Hz-wide pass band for the central frequency ranging from 4 to 28 Hz with a MATLAB function called eegfilt.m. The bandpass-filtered signal was detrended by subtracting the mean amplitude across the entire trial epoch to remove the DC noise. Next, the Hilbert transformation (Hilbert.m) was applied to the resultant detrended-filtered signal to estimate its instantaneous amplitude. Subsequently, the data for buffer periods on both ends of the experimental epochs were trimmed.

##### Statistical analysis

First, we selected the channels of interest based on previous studies. More specifically, for the frontal positivity, we examined the ERP response observed at channel Fz. For the occipital alpha power suppression, we examined the oscillatory power response in 9–13-Hz activity observed at channel O1/2, OL/R, and PO3/4 by creating the averaged channel (e.g., Friedman & Johnson, 2000; Hanslmayr et al., 2012; see Fig. 3 for confirmatory topography observed in our data). For the visually evoked N1(see the Results section in Experiment 1 for the post hoc analysis), we examined the negative deflection observed approximately 150 ms after the onset of the stimulus observed at channel O1/2, OL/R, and PO3/4 by creating the averaged channel (e.g., Luck, Woodman, & Vogel, 2000; Vogel & Luck, 2000). Although the temporal characteristics of these EEG signals are relatively well-established in the previous literature, it is also known that individuals vary in the exact timing to elicit a given EEG component. These individual differences make it difficult to apply a fixed time window across individuals to compute the magnitude of the EEG responses. Therefore, we employed a statistical procedure developed by Sawaki and colleagues (Sawaki, Geng, & Luck, 2012) that allowed us to have individually tailored measurement window around the commonly defined measurement window. Specifically, we first specified the time window of interest for each EEG measure (i.e., 200–1,000 ms for the frontal positivity, 500–1,000 ms for the occipital alpha power and 100–200 ms for the visually evoked N1). These wide time windows were intentionally chosen to encompass the individual differences in the exact timings of each EEG response. In the specified wide time windows, we then computed the area for which the predicted patterns of EEG responses were observed. For example, when we tested the prediction that the voluntary up-regulation of memory encoding is reflected in the enhanced frontal positivity, we calculated the area in which the frontal positivity amplitude for the up-regulation condition was larger than that for the neutral condition 200–1,000 ms after the stimulus onset for each individual. The individually tailored differences in the frontal positivity amplitudes were then averaged across individuals to calculate the mean magnitude of the effect of voluntary up-regulation on the frontal positivity. Next, in order to estimate the null distribution of this effect (i.e., the distribution of the magnitude of this effect obtained just by chance), we flipped the condition labels of the interest (e.g., up-regulation and neutral) for a randomly selected subset of individuals. Then, we calculated the individually tailored differences in the same manner to compute the mean magnitude of the effect of interest. This permutation procedure was repeated 100,000 times to estimate the null distribution of the effect. Lastly, the observed effect was nonparametrically compared against the null distribution. That is, if the observed effect was more extreme than the 5% predicted end of the null distribution, we rejected the null hypothesis. These permutation-based nonparametric statistical approaches have been proven effective in recent electrophysiological and neuroimaging studies (Maris & Oostenveld, 2007; Sawaki et al., 2012; Sawaki & Luck, 2013).

### Results

#### Behavioral results

**Fig. 2:**
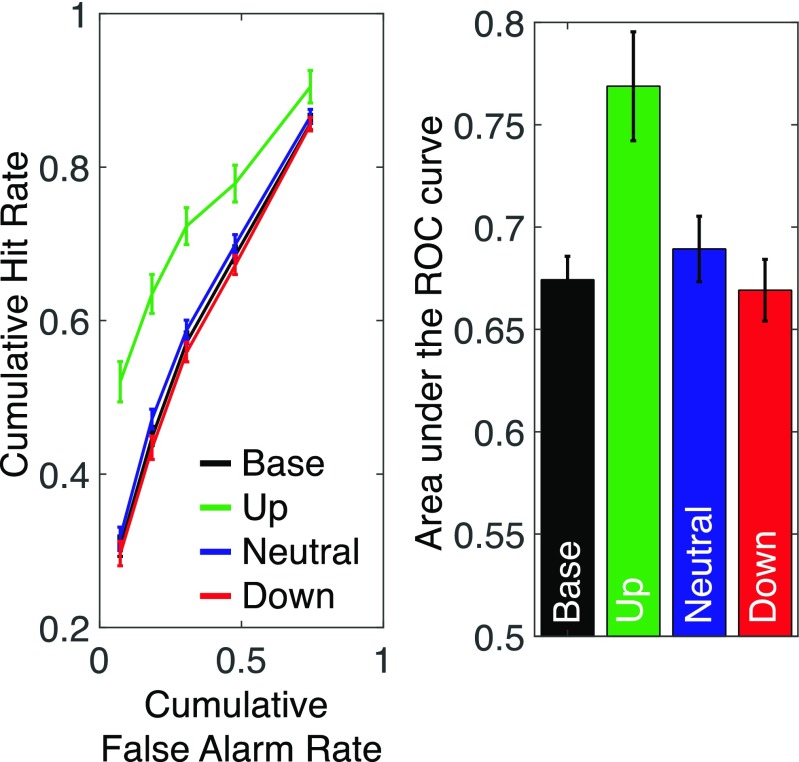
Behavioral results of Experiment 1. Left panel shows the ROC curves for each cue condition. Right panel shows the area under the ROC curves. Error bars indicate within-subject 95% confidence intervals

Table 1 shows the mean response proportions across six response types for all the conditions, from which the ROC curves were constructed. The ROC curves (see Fig. 2) show that memory was superior following up-regulation cues, but it did not differ among the other three conditions. The repeated-measures ANOVA confirmed a significant main effect of cueing conditions, *F*(3 ,69) = 19.69, *p* < .001, η_p_^2^ = .46. More precisely, the area under the ROC curve was significantly larger for up-regulation condition than the other three conditions, *t*(23) = 5.22, *p* < .001, scaled JZS Bayes factor favoring our hypothesis = 878.77 against baseline condition; *t*(23) = 4.06, *p* < .001, scaled JZS Bayes factor favoring our hypothesis = 66.35 against neutral condition; *t*(23) = 5.64, *p* < .001, scaled JZS Bayes factor favoring our hypothesis = 2,237.41, against down-regulation condition, and the area under the ROC curve for down-regulation condition was not worse than the baseline and neutral conditions, *t*(23) = 0.58, *p* = .57, JZS Bayes factor favoring the null hypothesis = 4.00 against the baseline condition; *t*(23) = 1.62, *p* = .12, JZS Bayes factor favoring the null hypothesis = 1.49 against the neutral condition. These results clearly suggest that participants successfully up-regulated memory encoding following the up-regulation cue while they failed to down-regulate memory encoding reliably following the down-regulation cue.

**Table 1:** Distribution of mean response proportions across conditions for Experiment 1

Condition	Response type					
	100% old	80% old	60% old	60% new	80% new	100% new
Baseline	0.31 (0.17)	0.14 (0.08)	0.12 (0.07)	0.12 (0.09)	0.18 (0.09)	0.14 (0.17)
Neutral	0.32 (0.18)	0.15 (0.09)	0.11 (0.07)	0.12 (0.09)	0.17 (0.11)	0.13 (0.17)
Up-regulation	0.52 (0.20)	0.12 (0.06)	0.09 (0.06)	0.06 (0.05)	0.13 (0.09)	0.09 (0.09)
Down-regulation	0.30 (0.12)	0.14 (0.08)	0.12 (0.08)	0.12 (0.10)	0.19 (0.09)	0.14 (0.16)
New	0.08 (0.09)	0.11 (0.08)	0.12 (0.07)	0.17 (0.14)	0.27 (0.11)	0.25 (0.21)

(values in the parentheses indicate the standard deviation of response proportions)

#### EEG results

**Fig. 3:**
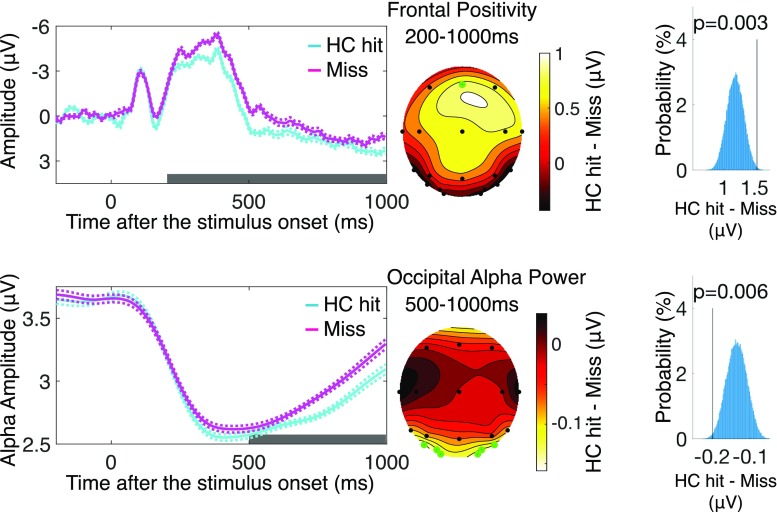
EEG results of Experiment 1. Column shows frontal positivity (top) and occipital alpha power (bottom) recorded during encoding task for high-confidence hit items (recognized with high confidence, or HC) and for missed items (not recognized). Error region represents within-subject standard error of the mean. Gray bars indicate the a priori defined measurement windows for each EEG correlate of memory encoding. Middle column shows topographical distribution of the subsequent memory effect (i.e., amplitude difference between HC hit and miss) for frontal positivity (top) and occipital alpha power (bottom). Green dots represent a priori determined channels of measurement. Right column shows result of nonparametric permutation-based statistical analysis for subsequent memory effect for frontal positivity (top) and occipital alpha power (bottom). Histogram represents null distribution derived from permutation procedure. Black line and *p* value indicate observed effect. (Color figure online)

Replicating previous results, we observed that the frontal positivity was larger for the items that were recognized with high confidence than those that were later missed (see Fig. 3, *p* = .003). This confirmed the validity of the frontal positivity as a measure of successful memory encoding. Next, we examined the occipital alpha power. Again, replicating previous findings, we observed that the occipital alpha power was weaker for the items that were later recognized with high confidence than those that were later missed (see Fig. 3, *p* = .006). This confirmed the validity of the occipital alpha power as a measure of successful memory encoding.

**Fig. 4:**
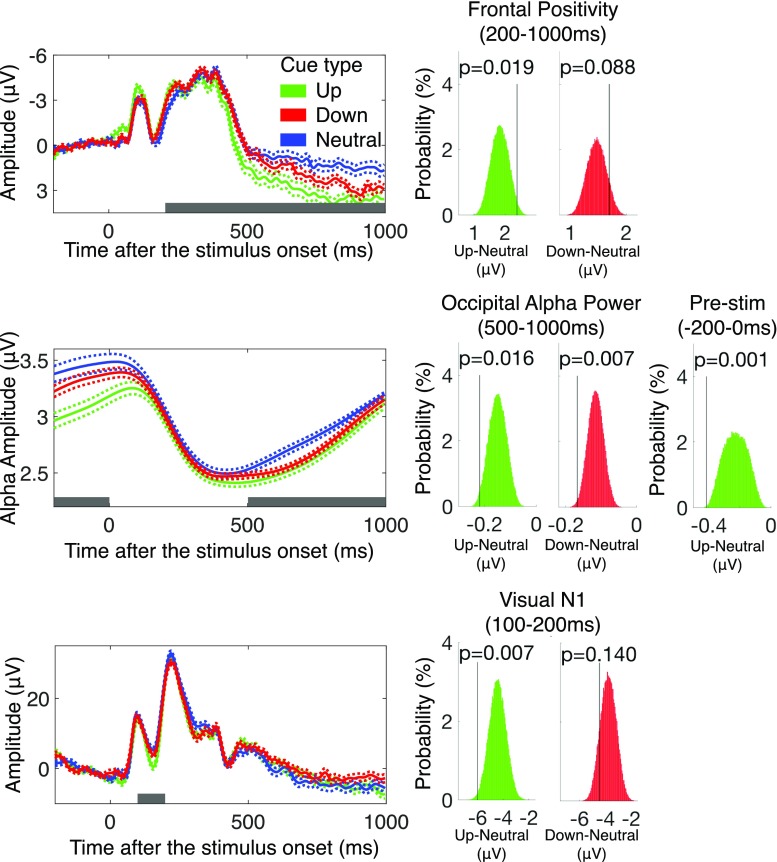
EEG Results of Experiment 1. Left column shows frontal positivity (top), occipital alpha power (middle), and visual N1 (bottom) recorded during encoding task for up-regulation (UP), down-regulation (Down), and neutral (Neutral) conditions. Error region represents within-subject standard error of the mean. Gray bars indicate the a priori defined measurement windows for each EEG correlate of memory encoding. Histograms show result of the nonparametric permutation-based statistical analysis for effect of voluntary control effect for frontal positivity (top), occipital alpha power (middle), and visual N1 (bottom). Green histograms represent null distribution derived from permutation procedure for effect of voluntary up-regulation, and the ed histograms show same for voluntary down-regulation. Black lines and *p* values indicate observed effects. Of note, observed effect of voluntary down-regulation is opposite in direction to what would be expected if voluntary down-regulation was possible. (Color figure online)

After verifying that the two neural correlates indexed memory encoding success in our data set, we then examined the effect of voluntary control on these correlates. Importantly, to control for any effect due to the physical onset of the cue, we compared the up-regulation and down-regulation condition against the neutral condition. As can be seen in Fig. 4, we found that the frontal positivity was larger in the up-regulation condition than in the neutral condition. Our nonparametric permutation test confirmed this observation (*p* = .019). Together with our behavioral results, this finding shows that we are capable of voluntarily up-regulating memory encoding. When we examined the effect of voluntary down-regulation of memory encoding, we found that the frontal positivity was, if anything, larger in the down-regulation condition than in the neutral condition (see Fig. 4, *p* = .088, for down-regulation > neutral). This result indicates that our participants did not suppress the frontal positivity to down-regulate our memory encoding, and therefore corroborates our behavioral finding.

Next, we examined the effect of voluntary control on the occipital alpha power suppression. As can be seen in Fig. 4, the occipital alpha power was significantly more reduced following the up-regulation cue than following the neutral cue (*p* = .016). When we examined the effect of voluntary down-regulation of memory encoding, we found that the occipital alpha power was, if anything, smaller following the down-regulation cue than the neutral cue (see Fig. 4, *p* = .007 for down-regulation < neutral). These results indicate that our participants successfully reduced the occipital alpha power to up-regulate memory encoding while they did not increase the occipital alpha power to down-regulate memory encoding, and therefore corroborate our behavioral findings.

Lastly, we examined if the observed voluntary control of memory encoding was the result of the front-end attentional allocation. In support of our hypothesis, we found that the occipital alpha power was more strongly suppressed even prior to the onset of the stimulus (i.e., 200-0 ms before the onset of the stimulus) following the up-regulation cue than following the neutral cue (*p* = .0006). This was not the case following the down-regulation cue (*p* = .15, *n..*). Based on previous findings that attentional prioritization of upcoming stimulus is reflected in prestimulus suppression of the occipital alpha power (e.g., Fukuda, Kang, & Woodman, 2016; Kelly, Gomez-Ramirez, & Foxe, 2009; O’Connell et al., 2009; Rohenkohl & Nobre, 2011; Sauseng et al., 2005; Thut, Nietzel, Brandt, & Pascual-Leone, 2006), our result demonstrates that participants successfully up-regulated the attentional allocation to the stimulus following the up-regulation cue, but did not down-regulate it following the down-regulation cue. To further confirm our interpretation, we conducted a post hoc analysis of the visually evoked N1 amplitudes. The visually evoked N1 is a negative deflection observed over parieto-occipital channels approximately 150 ms after the onset of visual stimulus, and its amplitude is magnified when the stimulus is attended and subjected to further cognitive processes beyond its detection (Luck et al., 2000; Vogel & Luck, 2000). If participants upregulated the attentional allocation following the up-regulation cues, we should expect that the N1 amplitude for the stimulus to be larger following the up-regulation cue than following the neutral cue. This was precisely the case (see Fig. 4, *p* = .008). When we examined the N1 amplitude following the down-regulation cue, it was statistically equivalent (numerically larger if anything) when compared with that following the neutral cue (Fig. 4, *p* = .13). These results demonstrate that the observed voluntary up-regulation of memory encoding was preceded by the successful modulation of the frontend attentional allocation.

### Discussion

In Experiment 1, we examined the nature and the extent of our ability to voluntarily regulate visual long-term memory encoding. Behavioral results clearly showed that we are capable of voluntarily up-regulating visual long-term memory encoding. The electrophysiological results showed that the magnitudes of the frontal positivity as well as the occipital alpha power suppression were larger for the up-regulation condition than for the neutral condition. These findings align nicely with the previous findings that the increased frontal positivity and the suppressed occipital alpha power index successful encoding into visual long-term memory. In addition, by examining the prestimulus occipital alpha power suppression and the visually evoked N1, we confirmed that this voluntary upregulation of memory encoding was preceded by successful allocation of attentional resources toward the to-be-up-regulated stimuli.

On the other hand, we failed to demonstrate our ability to voluntarily down-regulate visual long-term memory encoding. Both behavioral and electrophysiological results revealed that memory encoding was no worse following the *down-regulation* cue than following the *neutral* cue. If anything, the two EEG correlates of successful memory encoding were up-regulated following the down-regulation cue. These results show that our participants could not voluntarily down-regulate the memory encoding of these individual object pictures into visual long-term memory on a trial-by-trial basis.

## Experiment 2

The results of Experiment 1 clearly demonstrated that we are capable of voluntarily up-regulating visual long-term memory encoding. These results add to the previous demonstrations of reward-driven up-regulation of memory encoding by showing that exertion of such voluntary control does not require reward (Adcock et al., 2006; Gruber & Otten, 2010; Gruber et al., 2013). These findings are encouraging considering the amount of visual information that we need to encode into visual long-term memory in everyday life. However, it is unclear at this point if there is any negative side effect associated with the voluntary up-regulation of memory encoding, especially when we do so frequently in a limited amount of time with a limited amount of cognitive resource available (e.g., when cramming for a test the night before). Therefore, in Experiment 2, we examined if up-regulation of memory encoding for a cued item incurs any cost to memory encoding of other items, especially when there is not enough time and cognitive resource to encode all of the items. If there is none, to maximally enhance visual long-term memory encoding, we should simply provide the up-regulation cue following every item that needs to be encoded into visual long-term memory. On the other hand, if there is a cost associated with voluntary up-regulation of memory encoding, we should be doing so sparingly to maximize its benefit while minimizing its negative side effect. Therefore, in Experiment 2, we parametrically manipulated the cueing probability in a blocked manner to characterize the side effect of voluntary up-regulation of memory encoding.

### Method

#### Power calculation

In Experiment 2, we conducted a repeated-measures ANOVA with a 2 (cue probabilities: 20% and 50%) × 2 (cue: cued &and uncued) factorial design. Importantly, 0% and 100% cue probability conditions were tested using a separate paired-samples *t* test because, in these conditions, it was either cued (100%) or noncued (0%) items that were presented. Given that the effect size of the up-regulation cueing effect (i.e., the difference in the area under the ROC curves between up-regulation condition and baseline condition) was quite large (Cohen’s *d* = 1.06) in Experiment 1, we anticipated that the obtained effect size to be large (Cohen’s *d* = 1 or Cohen’s *f* = .5; Cohen, 1988). In addition, we found that the correlation between the area under the ROC curve for up-regulation condition and baseline condition was as high as .60 in Experiment 1. Based on these observations, the a priori power calculation with alpha level of .05 and the statistical power of .8 showed that we would need 10 participants for both the planned *t* test and the repeated-measures ANOVA (Faul et al., 2007). This assures that our sample size is sufficient to detect a large-size effect with .8 statistical power.

#### Participants

A different set of 32 participants from the same pool completed the study. The data from two participants were excluded from the analysis due to failure to comply with the task instructions.

#### Procedures

The encoding task was the same as for Experiment 1, except for the following. We only tested baseline and up-regulation conditions. Next, the cueing probabilities (0%, 20% 50%, and 100%) were manipulated in blocks. More precisely, in 0% cueing blocks, no items were preceded with the up-regulation cue. In 20% and 50% cueing blocks, a randomly selected 20% and 50% of the items were preceded by the up-regulation cue, respectively. In 100% cueing blocks, all the items were preceded by the up-regulation cue. Participants encoded 120 pictures per encoding block, and they completed two blocks for each cueing probability. The block order was randomly assigned to each participant. Finally, the recognition test was administered immediately following each encoding block. Each recognition block contained 60 old pictures (60 uncued items in 0% condition; 30 cued and 30 uncued items in 20% and 50% cueing condition; and 60 cued items in 100% cueing condition) and 30 new pictures. Of note, there were more studied pictures presented than new pictures during the recognition test, thus likely inducing response bias favoring “remember” responses across the board. However, this does not affect any cueing probability condition selectively, and therefore does not interfere with our interpretation of the effect of cueing probability.

To measure recognition performance, we constructed separate ROC curves for each encoding condition. Since participants were instructed to judge whether the presented item was presented anytime during the preceding encoding block, the ROC curves for cued and uncued items were constructed using the common false-alarm rates for the new items presented in the same recognition block.

### Results

#### Behavioral results

**Fig. 5:**
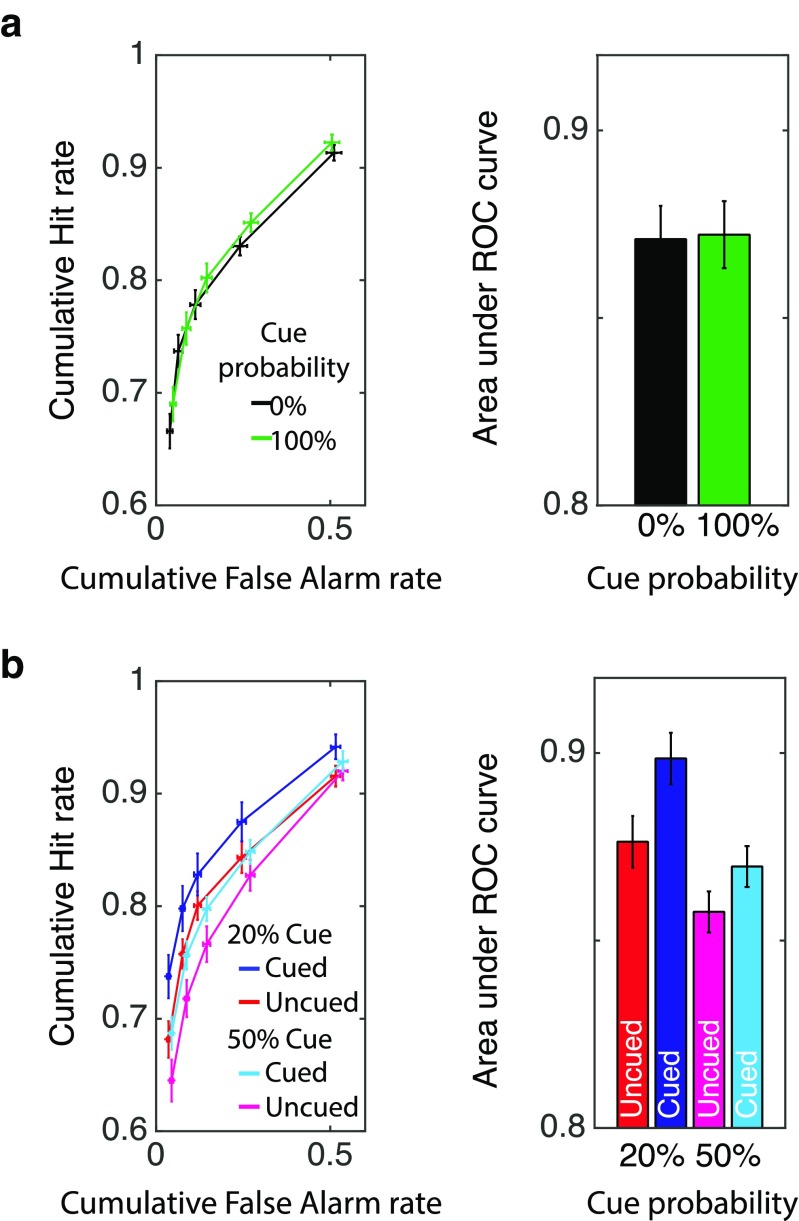
Behavioral results of Experiment 2. **a** Result for 0% and 100% cue probability conditions. **b** Result for 20% and 50% cue probability conditions. Left column shows ROC curves for each condition. Right panel shows corresponding area under ROC curve for each condition. Error bars indicate within-subject 95% confidence intervals

Table 2 shows the mean response proportions for six response types across all the conditions. As can be seen, the recognition performance for 0% cueing condition was clearly below ceiling, and therefore, it confirmed that participants did not have enough time and cognitive resource to encode all of the stimuli. We began our analyses by examining whether it is possible to voluntarily up-regulate memory encoding when all the items are cued. We compared the area under the ROC curves between the 0% cued condition and 100% cued condition. As Fig. 5 shows, there was no reliable difference between the two conditions, *t*(29) = 0.14, *p* = .90, scaled JZS Bayes Factor favoring the null hypothesis = 5.10. This result clearly indicates that participants failed to up-regulate memory encoding of cued items when all the items were cued. Next, we examined the performance on 20% and 50% cueing blocks. A repeated-measures ANOVA revealed that there was a significant main effect of cue, *F*(1, 29) = 13.47, *p* < .01, η_p_^2^ = .32, as well as a significant main effect of cue probability, *F*(1, 29) = 25.88, *p* < .01, η_p_^2^ = .47. The interaction between the cue and the cue probability was not significant, *F*(1, 29) = 1.39, *p* = .25, η_p_^2^ = .05. That is, in both cue probability conditions, the cued items were better recognized than uncued items to the same extent.

**Table 2:** Distribution of mean response proportions across conditions for Experiment 2

Cueing probability	Condition	Response type					
		100% old	80% old	60% old	60% new	80% new	100% new
0%	Uncued	0.67 (0.22)	0.07 (0.07)	0.04 (0.04)	0.05 (0.05)	0.08 (0.08)	0.09 (0.09)
	New	0.04 (0.05)	0.02 (0.03)	0.05 (0.07)	0.13 (0.12)	0.27 (0.15)	0.49 (0.26)
100%	Cued	0.69 (0.20)	0.07 (0.06)	0.05 (0.05)	0.05 (0.06)	0.07 (0.08)	0.08 (0.11)
	New	0.05 (0.06)	0.04 (0.04)	0.06 (0.07)	0.13 (0.12)	0.23 (0.17)	0.50 (0.29)
20%	Uncued	0.68 (0.20)	0.08 (0.07)	0.04 (0.04)	0.04 (0.05)	0.07 (0.08)	0.08 (0.12)
	Cued	0.74 (0.19)	0.06 (0.05)	0.03 (0.04)	0.05 (0.07)	0.07 (0.07)	0.06 (0.10)
	New	0.04 (0.04)	0.04 (0.05)	0.04 (0.04)	0.13 (0.11)	0.27 (0.15)	0.48 (0.26)
50%	Uncued	0.65 (0.21)	0.07 (0.06)	0.05 (0.04)	0.06 (0.09)	0.09 (0.08)	0.08 (0.11)
	Cued	0.69 (0.21)	0.07 (0.06)	0.04 (0.04)	0.05 (0.07)	0.08 (0.07)	0.07 (0.11)
	New	0.04 (0.04)	0.04 (0.05)	0.06 (0.05)	0.13 (0.11)	0.27 (0.13)	0.46 (0.24)

(values in the parentheses indicate the standard deviation of response proportions)

In addition, the recognition performance for both cued and uncued items were better in 20% condition than in 50% condition. These results indicate that although the memory encoding of cued items were voluntarily up-regulated when cues were provided, too frequent cueing (i.e., 50%) lowered the recognition performance for both cued and uncued items.

To characterize the effect of cueing probabilities on the cueing effect further, we have conducted a series of post hoc *t* tests. While the memory performance for the cued items in the 20% cueing condition was statistically higher than memory performance for cued items in the 50% and 100% cueing condition, *t*(29) = 4.12, *p* < .001, Bonferroni corrected, scaled JZS Bayes Factor favoring our hypothesis = 100.07 against 50% cueing condition; *t*(29) = 3.69, *p* = .01, Bonferroni corrected, scaled JZS Bayes Factor favoring our hypothesis = 35.61 against 100% cueing condition, memory performance for cued items in the 50% cueing condition was not higher than that for 100% cueing condition, *t*(29) = 0.43, *p* = .66, scaled JZS Bayes Factor favoring null hypothesis = 4.72. As for the memory performance for the uncued items, while that in the 20% cueing condition was not statistically different from that in the 0% cueing condition, *t*(29) = 0.76, *p* = .46*.*, scaled JZS Bayes Factor favoring null hypothesis = 3.94, it was statistically higher than that in 50% cueing condition, *t*(29) = 3.34, *p* < .01, Bonferroni corrected, scaled JZS Bayes Factor favoring our hypothesis = 15.86. The memory performance for uncued items in 50% cueing condition was numerically lower than that for 0% cueing condition, but it did not reach statistical significance, *t*(29) = 1.44, *p* = .16, scaled JZS Bayes Factor favoring null hypothesis = 2.03. Taken together, these results are in line with our interpretation that voluntary up-regulation of memory encoding is enabled by biasing the competition for the cognitive resource toward the encoding of the cued item at hand and away from the encoding of the other items. As a result, when cognitive resource for memory encoding is not sufficiently available, the other items that lose in the competition will be encoded more poorly.

### Discussion

The results of Experiment 2 demonstrated that there is a limit in our ability to voluntarily up-regulate visual long-term memory encoding when there is not enough time and cognitive resources to encode everything. More precisely, it was impossible to voluntarily up-regulate memory encoding of all items presented in the 100% cued blocks, and more importantly, too frequent voluntary up-regulation of cued items lowered the memory performance for cued as well as uncued items presented in the same block. These results show that voluntary up-regulation of memory encoding is the result of biased competition of cognitive resources toward the encoding of the cued items and away from the encoding of the other items, and therefore, there is a cost paid to voluntarily up-regulate memory encoding.

## General discussion

Voluntary control over the moment-to-moment fluctuations in our ability to encode information for future retrieval allows for the most adaptive use of our long-term memories. To test the efficacy of the voluntary regulation of memory encoding, we provided symbolic cues that instructed participants to either up-regulate or down-regulate memory encoding prior to perceptual encoding of the stimulus. Building on previous demonstrations that used reward to motivate memory encoding (Adcock et al., 2006; Gruber & Otten, 2010; Otten, Quayle, Akram, Ditewig, & Rugg, 2006), we found that we are capable of voluntarily up-regulating our memory encoding following the cue even without external reward, and it was reflected in the modulation of previously established neural correlates of memory encoding success, namely, the occipital alpha power suppression and the frontal positivity. In contrast, our behavioral and electrophysiological results showed that our participants failed to voluntarily down-regulate their memory encoding on a trial-by-trial basis. In Experiment 2, we further characterized the nature of our ability to voluntarily up-regulate memory encoding by manipulating the probability of the voluntary up-regulation. Here we found that, when cognitive resource is not sufficient to encode all of the items, voluntary up-regulation of memory encoding came with a cost such that too frequent up-regulation of memory encoding resulted in impaired memory encoding for both cued and uncued items. These results show that voluntary up-regulation of memory encoding is the result of biased competition of cognitive resources toward the encoding of the cued item at hand and away from the encoding of the other items.

### EEG correlates of voluntary control of memory encoding

Our EEG results showed that our ability to voluntarily up-regulate memory encoding is reflected in the occipital alpha power suppression and in the frontal positivity. Given our previous demonstration that the magnitudes of these two signals are not correlated during encoding (Fukuda & Woodman, 2015), our current results suggest that voluntary up-regulation of memory encoding recruits multiple neural mechanisms. The malleability of these signals is in line with the previous demonstration that these neural signals are sensitive to the depth of encoding processes (Fabiani, Karis, & Donchin, 1990; Fernández et al., 1998; Hanslmayr, Spitzer, & Bauml, 2009). More precise understanding of these two electrophysiological correlates, particularly with regard to their dissociability, would enlighten our understanding of the multifaceted nature of memory encoding processes.

One limitation of our study is that we selected the EEG signals of interest based on previous literature of memory encoding success, or namely the subsequent memory effect (Friedman & Johnson, 2000; Hanslmayr et al., 2012). We did so on purpose to make straightforward predictions about what should happen if bidirectional memory control were to be successfully exerted. However, this theory-driven approach could have limited our scope in finding dissociable EEG signals responsible for up- and down-regulation of memory encoding (Anderson & Hanslmayr, 2014). To properly look for such dissociable EEG signals, future studies need to first establish a reliable behavioral effect of down-regulation of memory encoding with sufficient statistical power to support the data-driven analyses.

### Asymmetric nature of voluntary control of memory encoding

Our results showed that although participants were capable of voluntarily up-regulating memory encoding, they were unsuccessful at voluntarily down-regulating it. Although it is difficult to claim that we are not capable of voluntarily down-regulating memory encoding at all, our results at least show the asymmetry in the ease of voluntary control of memory encoding. The lack of evidence for voluntary down-regulation of memory encoding might look inconsistent with recent demonstrations of active suppression of distracting information (e.g., Gaspelin & Luck, 2017). One important discrepancy between our paradigm and the paradigms used to demonstrate active suppression is the isolated presentation of cued-to-be-forgotten (or to-be-actively-suppressed) items. In studies that demonstrated active suppression of distractors, the to-be-suppressed items were always presented with other items (i.e., to-be-selected and/or neutral items). Even in such cases, some studies found that these to-be-suppressed items were first selected and then later suppressed (Cunningham & Egeth, 2016; Fukuda & Vogel, 2011; Moher & Egeth, 2012). Taken together, it would be interesting to see if the presentation of multiple items is necessary to down-regulate the memory encoding of to-be-forgotten items.

Next, our findings may also seem to run counter to a long line of research using directed-forgetting paradigms (Anderson & Hanslmayr, 2014; Bjork et al., 1998; Fawcett, Lawrence, & Taylor, 2016; Fawcett & Taylor, 2008). There are several important methodological features that we would like to highlight to reconcile the differences. First, our study, unlike the majority of directed forgetting research, included a baseline condition in which memory encoding was not up-regulated or down-regulated. A few recent studies that did include a within-subject baseline condition akin to ours (Gao et al., 2016; Zwissler et al., 2015) have found results consistent with ours such that the memory performance for cued-to-be-forgotten items were no worse than that for baseline items. Collectively, our findings indicate that voluntary down-regulation of memory encoding is at least much more difficult, if not impossible, than voluntary up-regulation of memory encoding when such cognitive control is required on a trial-by-trial basis, and this voluntary up-regulation of memory encoding for cued-to-be-remembered items can account for the difference in memory performance between cued-to-be-remembered items and cued-to-be-forgotten items. This interpretation is generally in line with the selective rehearsal account of item-based-directed forgetting (Hourihan, Ozubko, & MacLeod, 2009; MacLeod, 1998). According to this account, item-wise directed forgetting is not the result of active suppression of memory encoding of to-be-forgotten items, but of differential allocation of resources for memory encoding favoring the cued-to-be-remembered items over the cued-to-be-forgotten items.

Second, unlike our experiments, typical item-method directed-forgetting studies utilize postcue paradigms in which the cues to remember or to forget are presented after the stimuli. This includes the recent studies that found that the memory performance for cued-to-be-forgotten items were not worse than that for baseline items. Thus, our findings using precues make a unique contribution by demonstrating that our inability to voluntarily down-regulate memory encoding is not limited to the situation when the memory control is exerted after perceptual encoding of the stimulus. In fact, our findings demonstrated this inability even when the down-regulatory memory control was summoned one second prior to the perceptual encoding of the stimulus. Previous studies in attentional control literature suggest a potential mechanism for this cognitive inflexibility. Recent studies that examined our ability to actively suppress task-irrelevant distractors have shown that in order for us to exert such control effectively, to-be-suppressed information have to stay consistently predictable over a long period of time (Cunningham & Egeth, 2016; Moher & Egeth, 2012; Noonan et al., 2016; Noonan et al., 2017). If active attentional suppression underlies the down-regulation of memory encoding, one can potentially facilitate it by presenting the cued-to-be-forgotten items consistently and predictively over a long period of time (e.g., blocking of to-be-forgotten items, or presenting them consecutively in a row). To evaluate its effectiveness, future studies need to compare its memory performance to the baseline items but not to the to-be-remembered items.

Third, it is also possible that down-regulation of memory is so effortful that it requires much more time than up-regulation (Cheng, Liu, Lee, Hung, & Tzeng, 2012; Fawcett & Taylor, 2008). Indeed, typical item-method directed-forgetting studies provide longer durations (up to several seconds) after the stimulus presentation for this cognitive processes to be complete (Fawcett et al., 2016; Fawcett & Taylor, 2008). Future studies should examine if down-regulation of memory encoding is possible even when much more time is provided between the precue and the to-be-remembered stimulus (but see also Bancroft, Hockley, & Farquhar, 2013). If individuals are capable of down-regulating memory encoding below baseline when provided with much longer time, our current findings still demonstrate that voluntary down-regulation of memory encoding, unlike up-regulation, cannot be exerted promptly when a cue indicates task relevant.

Lastly, one recent item-method directed-forgetting study found that to-be-forgotten items (i.e., items that were followed by the *forget* cue) were remembered more poorly than the baseline items established in a within-subject manner were (Taylor et al., 2018). Furthermore, they also found that the to-be-remembered items (i.e., items followed by the *remember* cue) that were presented randomly intermixed with to-be-forgotten items were not remembered better than the baseline items. Although this finding seems to contrast to our findings as well as others (Gao et al., 2016; Zwissler et al., 2015), there are two notable differences in the experimental paradigms.

First, in their study, the within-subject baseline was established by assessing participants’ memory for items presented in blocks, in which participants were instructed to remember all the items. Thus, there was no instructional difference between the to-be-remembered items and the baseline items. This makes it difficult to assess whether their participants attempted to encode stimuli any differently between the two conditions. If they attempted to encode both stimuli equally well and the experimental paradigm allowed sufficient time to encode both stimuli well, it may not be too surprising to see no difference in memory performance between the two conditions.

Second, participants were given much longer time to view the stimuli (i.e., 1,000 ms stimulus presentation as opposed to 250 ms in both of our experiments). This long viewing time likely allowed for sufficient memory encoding for the baseline items presented in the blocks with all relevant items, and therefore left little room for memory up-regulation for the cued-to-remember items presented intermixed with the cued-to-forget items. Indeed, the average hit rate reported in Taylor et al. (2018) for the baseline items (84%) was considerably higher than what we observed for the baseline items in Experiment 1 (57%), while the false alarm for the baseline items in their study (7%) was much lower than what we observed in Experiment 1 (31%). Moreover, their memory performance for the baseline items was numerically higher than the highest performance that we observed in up-regulation conditions across both of our experiments (i.e., up-regulation condition in Experiment 2: the average hit rate = 83% and the average false-alarm rate = 16%). Although this comparison needs to be interpreted cautiously due to many methodological differences between the studies, it nonetheless poses a possibility that memory encoding for the baseline items in Taylor et al. (2018) was already up-regulated due to ample viewing time, and that there was no room for improvement to be seen for cued-to-remember items presented with cued-to-forget items. If this was the case, lower memory performance for cued-to-forget items than for the baseline items in Taylor et al. (2018) could be the result of superb or up-regulated memory performance for the baseline items. Future studies should directly investigate this possibility.

### Negative side effect of voluntary up-regulation of memory encoding

We also found that too frequent voluntary up-regulation of memory encoding led to overall decrease in recognition performance in Experiment 2 when participants did not have sufficient cognitive resource to encode all of the stimuli well. In fact, asking participants to voluntarily up-regulate all the items led to the same performance as asking them to not voluntarily up-regulate any item. These findings have potential practical implications. For example, although we are capable of voluntarily up-regulating memory encoding of cued visual information (e.g., highlighted section in a textbook), such strategy has to be used with caution in order to avoid the costs that occur when encoding all items are deemed relevant (e.g., highlighting most of a page of a book) especially when cognitive resource available for memory encoding is limited. In other words, it is important to keep in mind the net effect on learning whenever one relies on the voluntary up-regulation of memory encoding. At the same time, it might also be possible to harness this negative side effect of too-frequent up-regulation of memory encoding to our benefit when we encounter information that we would like to not remember. More specifically, our findings suggest that when we unfortunately encounter some information that we wish not to remember, it might be possible to reduce its likelihood of later remembrance by voluntarily up-regulating the memory encoding of other information. Future studies should examine this possibility by directly manipulating the sequence of the up-regulation and baseline items.
